# Identification and VIGS-based characterization of *Bx1* ortholog in rye (*Secale cereale* L.)

**DOI:** 10.1371/journal.pone.0171506

**Published:** 2017-02-24

**Authors:** Jolanta Groszyk, Mariusz Kowalczyk, Yuliya Yanushevska, Anna Stochmal, Monika Rakoczy-Trojanowska, Waclaw Orczyk

**Affiliations:** 1 Department of Genetic Engineering, Plant Breeding and Acclimatization Institute – National Research Institute, Blonie, Poland; 2 Department of Biochemistry and Crop Quality, Institute of Soil Science and Plant Cultivation State Research Institute, Pulawy, Poland; 3 Department of Plant Genetics, Breeding and Biotechnology, Warsaw University of Life Sciences, Warsaw, Poland; University of Western Sydney, AUSTRALIA

## Abstract

The first step of the benzoxazinoid (BX) synthesis pathway is catalyzed by an enzyme with indole-3-glycerol phosphate lyase activity encoded by 3 genes, *Bx1*, *TSA* and *Igl*. A gene highly homologous to maize and wheat *Bx1* has been identified in rye. The goal of the study was to analyze the gene and to experimentally verify its role in the rye BX biosynthesis pathway as a rye ortholog of the *Bx1* gene. Expression of the gene showed peak values 3 days after imbibition (dai) and at 21 dai it was undetectable. Changes of the BX content in leaves were highly correlated with the expression pattern until 21 dai. In plants older than 21 dai despite the undetectable expression of the analyzed gene there was still low accumulation of BXs. Function of the gene was verified by correlating its native expression and virus-induced silencing with BX accumulation. *Barley stripe mosaic virus* (BSMV)-based vectors were used to induce transcriptional (TGS) and posttranscriptional (PTGS) silencing of the analyzed gene. Both strategies (PTGS and TGS) significantly reduced the transcript level of the analyzed gene, and this was highly correlated with lowered BX content. Inoculation with virus-based vectors specifically induced expression of the analyzed gene, indicating up-regulation by biotic stressors. This is the first report of using the BSMV-based system for functional analysis of rye gene. The findings prove that the analyzed gene is a rye ortholog of the *Bx1* gene. Its expression is developmentally regulated and is strongly induced by biotic stress. Stable accumulation of BXs in plants older than 21 dai associated with undetectable expression of *ScBx1* indicates that the function of the *ScBx1* in the BX biosynthesis is redundant with another gene. We anticipate that the unknown gene is a putative ortholog of the *Igl*, which still remains to be identified in rye.

## Introduction

Benzoxazinoids (BXs) are a group of defense-related molecules accumulated as glycosylated precursors in grasses and sporadically in a few dicot species [1]. The first reports published in the 1950s identified the BX as components of rye resistance against *Fusarium* [2] and maize resistance to the European corn borer *Ostrinia nubilalis* [3]. Further studies confirmed the diverse roles of BX in innate immunity against pests and fungal diseases in maize [4, 5], wheat [6] and rye [7]. On the other hand, degradation (i.e. detoxification) of the benzoxazinoids was found to be a pathogen strategy to overcome the chemical defense of the host [8]. Studies by Kettle *et al*. (2015) and Schulz *et al*. (2013) [9, 10] indicated that BX were responsible for rye allelopathy. Nutritional investigations revealed that the BXs themselves or the BX-derived compounds found in rye or wheat food products show potentially health-promoting activities [11]. The diverse spectrum of biological functions as well as the possible nutritional values caused that the BXs became a widely studied group of secondary metabolites in plants.

The pathway found in maize is composed of 10 enzymes (BX1 to BX10) converting sequentially indole-3-glycerol phosphate to 4-hydroxy-7-methoxy-2-[(3R,4S,5S,6R)-3,4,5-trihydroxy-6-(hydroxymethyl)oxan-2-yl]oxy-1,4-benzoxazin-3-one (DIMBOA-Glc) and 4,7-dimethoxy-2-{[3,4,5-trihydroxy-6-(hydroxymethyl)oxan-2-yl]oxy}-3,4-dihydro-2H-1,4-benzoxazin-3-one (HDMBOA-Glc) [12, 13]. The first step is indole synthesis catalyzed by indole-3-glycerol phosphate lyase (IGL) encoded by *Bx1*. The subsequent steps are catalyzed by cytochrome P450 monooxygenases encoded by the *Bx2*–*Bx5* genes [14]. The remaining genes are members of the CYP71 family: *Bx6* encoding 2-oxoglutarate dependent dioxygenase, *Bx7* encoding 7-O-methyltransferase, *Bx8* encoding UDP-glucosyltransferase and *Bx9* glucosylase [15, 16]. Recently Meihls et al. reported conversion of DIMBOA-Glc to HDMBOA-Glc in the reaction catalyzed by O-methyltransferases encoded by *Bx10a-c* genes [13]. The *Bx1*–*Bx5* genes were first identified in maize [14, 15, 17] and later in wild barley *Hordeum lechleri* (Steud.); *HlBx1*–*HlBx5* [18], wild diploid wheat *Triticum boeoticum* (Boiss.); *TbBx1*–*TbBx5* [19], hexaploid wheat *T*. *aestivum* (L.) (*TaBx1A*–*TaBx5A*, *TaBx1B*–*TaBx5B*, *TaBx1D*–*TaBx5D*) [20–22] and rye *Secale cereale* (L.) [23]. The maize genes *ZmBx1*–*ZmBx8* were found to be clustered and located on the short arm of the maize chromosome 4. In hexaploid wheat and diploid rye the *Bx* genes exist as small clusters which are located on four chromosomes. *Bx1* and *Bx2* are on chromosomes 4A, 4B, 4D in wheat and 7R in rye, *Bx3*–*Bx5* are on chromosomes 5A, 5B, 5D in wheat and 5R in rye [21, 24].

Characterization loss-of-function phenotypes is one of the main strategies to study biological function of a gene known only from its nucleotide sequence. Virus induced gene silencing (VIGS) is a method of choice to induce silencing of a target gene and it is particularly suitable for species with no collections of knock-out mutants and no methods of genetic transformation. The technique is based on targeted post-transcriptional gene silencing (PTGS) in host species activated by introduction a viral vector bearing a fragment of the target gene. It was first developed in dicots [25] and by constructing vectors derived from *Barley Stripe Mosaic Virus* (BSMV) the method was adapted for barley [26] and later for wheat [27], *Brachypodium distachyon* [28] and oat [29]. The method can be used to silence a single gene or simultaneously two different genes [30] and can be applied to genes expressed in root, leaves [31], spikes and grains [32]. VIGS approach has been used to verify gene function in biochemical pathway of morphine biosynthesis [33]. For a recent comprehensive review on VIGS see [34]. Designing the VIGS experiments and adapting the method for a new species the use of internal silencing standard is required. This is achieved by using a vector bearing a fragment of gene encoding phytoene desaturase (PDS), which is a key enzyme of carotenoid synthesis. Its silencing inhibits synthesis of carotenoids and leads, as a consequence, to photobleaching of chlorophyll. This is easily visible as white leaf sectors of the experimental plants. Silencing of the *PDS* and the presence of white leaf sectors serves as a visual marker confirming correctly constructed vectors and successful inoculation. Rye belongs to so called recalcitrant species for which transformation methods are practically not available, there are no mutant collections, rye genome has not been sequenced and there are very limited resources on genomic sequences of rye genes. In such species VIGS is a method of choice to characterize biological function of a specific gene. Rye is an important cereal used as grain, forage and a green manure crop. Less demanding growth conditions combined with frost and drought resistance make it suitable for the colder regions and poor soils of Northern and Eastern Europe, North America and Northern Asia. Rye is among the first species where benzoxazinoids have been identified; however, the genes involved in the BX biosynthesis pathway remain unknown until recently. The first two, *ScBx1* and *ScBx2*, were proposed by La Hovary in his PhD thesis [35]; however, the structure and the nucleotide sequences of the subsequent five genes–*ScBx1*, *ScBx2*, *ScBx3*, *ScBx4* and *ScBx5* –were published by Bakera *et al*. [23]. The two genes *Bx6* and *Bx7* encoding 7-hydroxylase and 7-O-methylotranferase, and converting 2,4-dihydroxy-1,4-benzoxazin-3-one (DIBOA-Glc) to (DIMBOA-Glc) in maize, have not been found in wheat or rye, although the same reactions are expected to occur in these species. However, the *Bx6*-*like* gene encoding DIBOA-Glc dioxygenase has been described in rye (GenBank: HG380520.1).

Conversion of indole-3-glycerol phosphate (IGP) to indole, catalyzed by indole-3-glycerol phosphate lyase (IGL), is the branching point between two pathways: BX synthesis and tryptophan synthesis. Proteins with IGL activity are encoded by three different genes: *Bx1*, *TSA* or *Igl* [36]. Although the genes are evolutionarily related and show high sequence homology, they differ in their expression patterns and, more importantly, they have different enzyme characteristics. BX1 (encoded by *Bx1*) acts as a monomer and delivers free indole, which is a substrate in the BX biosynthesis pathway [36]. The tryptophan synthase alpha (TSA) subunit is active only in a complex with the tryptophan synthase beta (TSB) subunit, and the catalyzed reaction provides indole directly to the tryptophan synthesis pathway [36, 37]. The third protein with IGL activity is encoded by the *Igl* gene. The enzyme is active as a monomer and in this respect can replace the BX1 enzyme in providing free indole to the BX pathway [14, 18, 22, 36]. The two genes involved in BX synthesis, i.e. *Bx1* and *Igl*, have different expression patterns. *Bx1* is developmentally regulated, while *Igl* is mostly herbivory-induced [4, 36]. The cDNA clones identified in rye were annotated as *Bx* biosynthesis genes mostly based on the sequence homology to already known genes [23].

Considering high homology of *Bx1* to *TSA*, to *TSA-like* as well as to *Igl*, the correct annotation of the identified putative *Bx1* gene in rye required experimental verification. This was the aim of the present study. The strategy of functional analysis of the gene was based on correlation of its native expression pattern and experimental VIGS-induced silencing with the levels of benzoxazinoids in rye leaves.

## Results

### *ScBx1* shows a strong, developmentally dependent expression pattern

**Fig 1:**
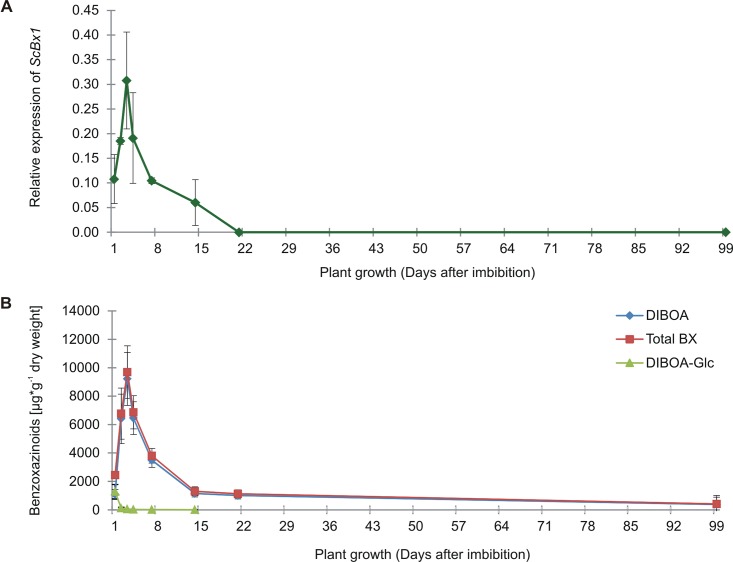
Relative level of *ScBx1* transcript and amount of benzoxazinoids in rye. Relative level of *ScBx1* transcript (**A**) and total amount of DIBOA/DIBOA-Glc and total amount of tested benzoxazinoids (**B**) in hypocotyls/leaves of rye cv. Stach F1 collected 1, 2, 3, 4, 7, 14, 21 and 99 days after imbibition. The results represent the mean values and the standard deviations from three biological replicates. *—total amount of: HBOA, DIBOA, DIBOA-Glc, DIMBOA, DIMBOA-Glc and MBOA.

Expression pattern of *ScBx1* in rye leaves indicated strong, developmentally dependent regulation. Relative levels of *ScBx1* transcript in hypocotyls collected 1, 2 and 3 dai were 0.1077 ± 0.0496, 0.1852 ± 0.0073 and 0.3078 ± 0.0983 respectively. In leaves sampled 4, 7, and 14 dai the amount of transcript was 0.1910 ± 0.0920, 0.1045 ± 0.0059 and 0.0602 ± 0.0464 respectively. Starting from 21 dai until the last tested time-point, i.e. 99 dpi, the transcript of the analyzed *ScBx1* was undetectable (Fig 1A). Out of the six tested benzoxazinoids– 2-hydroxy-4H-1,4-benzoxazin-3-one (HBOA), 2,4-dihydroxy-1,4-benzoxazin-3-one (DIBOA), DIBOA-Glc, 2,4-dihydroxy-7-methoxy-1,4-benzoxazin-3-one (DIMBOA), DIMBOA-Glc and 6-methoxy-3H-1,3-benzoxazol-2-one (MBOA)–the levels of the two compounds DIBOA and DIBOA-Glc were the highest at all tested time-points 1, 2, 3, 4, 7, 14, 21 and 99 dai (Fig 1B, S1 Table). On average the total amount of both DIBOA forms constituted over 93% of the total analyzed BX. The highest amount of all BX and the highest amount of DIBOA + DIBOA-Glc were detected in hypocotyls 3 dai (Fig 1B, S3 Table). *ScBx1* transcript levels and total BX concentrations in plant material are highly correlated (r = 0.83; N = 24; p < 0.05) (S1 Fig).

### The BSMV-VIGS system is suitable for target gene silencing in rye

**Fig 2:**
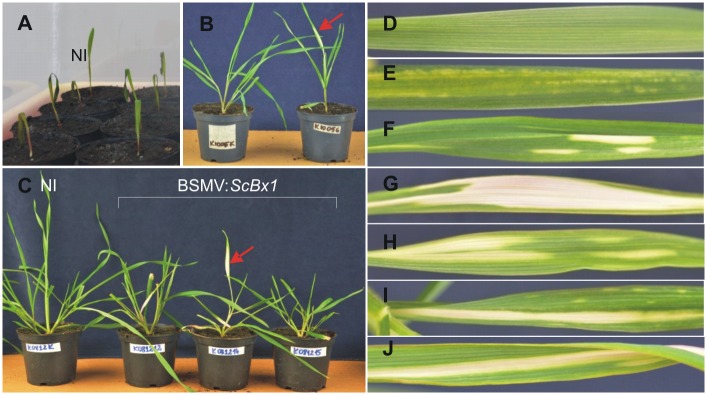
Rye seedlings used for inoculation and symptoms of *PDS* silencing. Rye seedlings 5 days after imbibition used for inoculation with the BSMV-derived vectors (**A**). Rye seedlings 14 days post inoculation (**B**) and 21 dpi (**C**). Control plant leaf (**D**), symptoms of BSMV infection (**E**), symptoms of *PDS* silencing (**F**-**J**).

Rye is a nonhost for *Barley stripe mosaic virus*; however, some of the rye seedlings inoculated with BSMV-based vectors developed symptoms of virus infection and, when the vector contained the *PDS* fragment, the seedlings showed symptoms of *PDS* silencing (Fig 2). Out of the 142 seedlings inoculated with the control vectors, i.e. BSMV:α,β_(-)_,γ_(-)_, BSMV:α,β_(-)_,γ_(*PDS*)_ or BSMV:α,β_(*PDS*)_,γ_(*PDS*)_, 13 seedlings developed symptoms of *PDS* silencing and/or virus infection (Table 1). Out of the 310 rye seedlings inoculated with the experimental vectors, i.e. BSMV:α,β_(*Bx*)_,γ_(*PDS*)_ and BSMV:α,β_(*PDS*)_,γ_(*Bx*)_, 31 seedlings developed symptoms of *PDS* silencing and/or virus infection (Table 1, Fig 2).

**Table 1:** Number of rye plants inoculated with VIGS-BSMV control vectors (BSMV:*PDS*) and vectors designed for PTGS containing cDNA fragment of *ScBx1* (BSMV:*ScBx1*).

Vector Name	Vector design	Number of inoculated plants	Number of plants with symptoms of *PDS* silencing	Inoculation efficiency [%]
BSMV:*PDS*	α,β_(-)_,γ_(-)_	142	13	9.1
	α,β_(-)_,γ_(*PDS*)_			
	α,β_(*PDS*),_γ_(*PDS*)_			
BSMV:*ScBx1*	α,β_(*Bx*)_,γ_(*PDS*)_	192	20	10.4
	α,β_(*PDS*)_,γ_(*Bx*)_	118	11	9.3

### Inoculation of rye seedlings with BSMV-VIGS vectors activates expression of *ScBx1* and stimulates accumulation of benzoxazinoids

**Fig 3:**
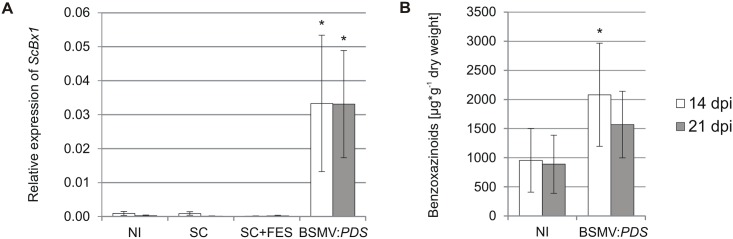
Relative expression of *ScBx1* and content of benzoxazinoids in control plants used for PTGS experiments. Relative expression of *ScBx1* (**A**) and content of benzoxazinoids (**B**) in non-inoculated leaves and in leaves inoculated with the control vector BSMV:α,β_(-)_,γ_(*PDS*)_ (BSMV:*PDS*). NI—non-inoculated seedlings, SC—leaves abraded with silicon carbide, SC+FES—leaves abraded with silicon carbide in FES buffer. BSMV:*PDS*—leaves abraded with silicon carbide and inoculated with BSMV:α,β_(-)_,γ_(*PDS*)_ vector suspended in FES. The results represent the mean value and standard deviation from three biological replicates. *–statistical significance (Student’s *t*-test) p < 0.05.

The relative expression of *ScBx1* in non-inoculated rye leaves was very low: 0.0009 ± 0.0007 and 0.0003 ± 0.0002 quantified 14 dpi and 21 dpi respectively. Similar low levels were detected in control leaves abraded with silicon carbide 0.0009 ± 0.0005 (14 dpi) and 0.0000 (21 dpi) and in mock-transfected control leaves abraded with silicon carbide in the presence of FES buffer 0.0000 (14 dpi) and 0.0002 ± 0.0002 (21 dpi). The relative expression of *ScBx1* in leaves inoculated with control vector carrying no insert BSMV:α,β_(-)_,γ_(*PDS*)_ labeled as BSMV:*PDS* was 0.0333 ± 0.0201 (14 dpi) and 0.0331 ± 0.0158 (Fig 3A).

The content of benzoxazinoids in leaves of non-inoculated rye seedlings was 955 ± 545 μg∙g^-1^ d.w. and 889 ± 499 μg∙g^-1^ d.w. 14 and 21 dpi respectively. The content of hydroxamic acids in rye leaves inoculated with the control BSMV:*PDS* vector, was 2081 ± 885 μg∙g^-1^ d.w. and 1570 ± 571 μg∙g^-1^ d.w. in leaves collected 14 and 21 dpi respectively (Fig 3B). Inoculation of rye seedlings with the control BSMV vector led to significant, over 40-fold induction of *ScBx1* expression (Fig 3A). This change was significant with higher content of benzoxazinoids (Fig 3B).

### A. Post-transcriptional silencing of *ScBx1* correlates with lower BXs content

**Fig 4:**
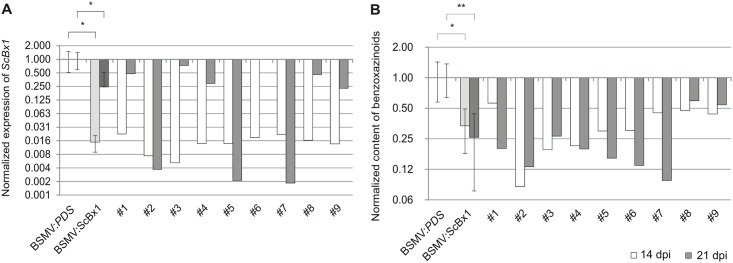
Normalized expression of *ScBx1* and normalized content of benzoxazinoids in PTGS experiments. Normalized expression of *ScBx1* (**A)** and normalized content of benzoxazinoids (BXs) (**B**) in the experimental plants (inoculated with BSMV:*ScBx1*) compared to the control (inoculated with BSMV:*PDS*). Results are shown on the logarithmic scale. BSMV:*PDS*–the mean value of the *ScBx1* relative expression in leaves inoculated with the control BSMV:α,β_(-)_,γ_(*PDS*)_ vector assumed as 1.00. BSMV:*ScBx1* –the mean value of *ScBx1* normalized expression in leaves inoculated with BSMV:*ScBx1* silencing vector. #1 –#9 –the *ScBx1* normalized expression in leaves of individual plants inoculated with the BSMV:*ScBx1* silencing vector. *–statistical significance (Student’s *t*-test): ** p < 0.005, * 0.005 < p < 0.05.

Inoculation of rye seedlings with BSMV-based control vectors showed that expression of the analyzed gene and the BX content was significantly enhanced compared with non-treated plants. This indicated that inoculation with the BSMV-based vectors activated two contradictory processes: up-regulation of the analyzed gene, which was the reaction for virus infection (Fig 3A), and gene silencing, which was induced by the BSMV-based VIGS vector. On average *ScBx1* normalized expression in plants inoculated with the experimental vector (BSMV:*ScBx1*) compared to the control (BSMV:*PDS*) was 62.5 x lower at 14 dpi and 4 x lower at 21 dpi. The strongest impact of the BSMV:*ScBx1* vector on *ScBx1* expression was in plants #2, #5 and #7 (Fig 4A).

The normalized content of BXs in plants inoculated with experimental vectors (BSMV:*ScBx1*) compared to the control (BSMV:*PDS*) was 3 x lower at 14 dpi and 3.9 x lower at 21 dpi (Fig 4B). The strongest impact of *ScBx1* silencing on BXs content was in plants #2, #3, #4, #5, #6 and #7 (Fig 4B). Correlation coefficient between normalized expression of *ScBx1* and the content of BXs in rye leaves inoculated with BSMV-VIGS vector and collected at 14 dpi was 0.85 (p = 0.008) (S2 Fig) and at 21 dpi was 0.57 (p = 0.13) (S3 Fig).

### B. Transcriptional gene silencing

**Fig 5:**
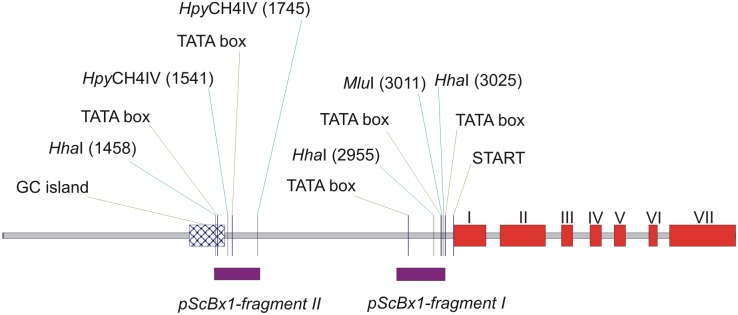
Schematic representation of gDNA (5029 bp) containing *ScBx1*. Indicated are: GC-islands and TATA-box motifs, start codon, exons, introns and the two gDNA fragments cloned to the VIGS-BSMV vector for transcriptional gene silencing (*pScBx1-fragment I* and *pScBx1-fragment II*). The sites recognized by methylation-sensitive restriction enzymes–*Mlu*I, *Hha*I and *Hpy*CH4IV—located within the cloned fragments of the *pScBx1* promoter region are indicated. The intron-exon structure of the *ScBx1* gene is based on [23].

Another series of VIGS-BSMV experiments was designed to induce transcriptional silencing (TGS) of the analyzed gene. The experimental vector BSMV:α,β_(*pScBx1-fragment I*)_,γ_(*pScBx1-fragment II*)_ marked as BSMV:*pScBx1* with gDNA fragments derived from the promoter region of *ScBx1* and the control vector BSMV:α,β_(-)_,γ_(-)_ marked as BSMV:00 were used for seedling inoculation (S2 Table, Fig 5). Out of 117 inoculated plants 17 plants developed symptoms of virus infection after inoculation with the control vector and 3 plants after inoculation with the experimental vector (Table 2). The 3 plants were analyzed for the *ScBx1* expression, cytosine methylation within the *ScBx1* promoter fragments and the content of hydroxamic acids. The leaf samples for the analysis were taken 14, 21 and 99 dpi.

**Table 2:** Number of rye plants inoculated with VIGS-BSMV control vectors (BSMV:00) and vectors designed for TGS containing promoter fragments of *ScBx1* (BSMV:*pScBx1*).

Vector Name	Vector design	Number of inoculated plants	Number of plants with symptoms of *PDS* silencing	Inoculation efficiency [%]
BSMV:00	α,β_(-)_,γ_(-)_	52	17	32.7
BSMV:*pScBx1*	α,β_(*pScBx1-fragment I*)_,γ_(*pScBx1-fragment II)*_	65	3	4.6

**Fig 6:**
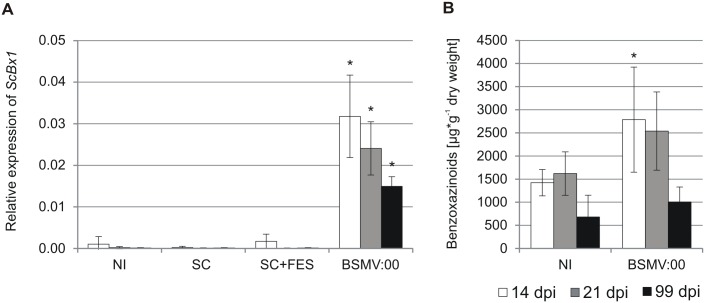
Relative expression of *ScBx1* and content of benzoxazinoids in control plants used for TGS experiments. Relative expression of *ScBx1* (**A**) and content of benzoxazinoids (**B**) 14, 21 and 99 days post inoculation (dpi) in plants not inoculated (NI), abraded with silicon carbide (SC), abraded with silicon carbide in the presence of FES buffer (SC+FES) and in plants inoculated with the ‘empty’ vector BSMV:α,β_(-)_,γ_(-)_ (BSMV:00). The results represent mean values and standard deviations from at least three biological replicates. *–statistical significance (Student’s *t*-test) p < 0.05.

Relative expression of *ScBx1* was very low in non-inoculated plants and in plants abraded with silicon carbide without or with FES buffer. Inoculation with BSMV:00 vector led to over 40-fold induction of *ScBx1* relative expression, which reached the levels 0.032 (14 dpi), 0.024 (21 dpi) and 0.015 (99 dpi) (Fig 6A).

Benzoxazinoid contents in leaves of non-inoculated plants were 1422, 1619 and 682 μg∙g^-1^ fresh weight 14, 21 and 99 dpi respectively. The BX contents in plants inoculated with the BSMV:00 vector were 2787, 2539 and 1003 μg∙g^-1^ fresh weight 14, 21 and 99 dpi respectively (Fig 6B).

### *ScBx1* promoter fragments in plants inoculated with BSMV:*pScBx1* vector show elevated cytosine methylation

**Fig 7:**
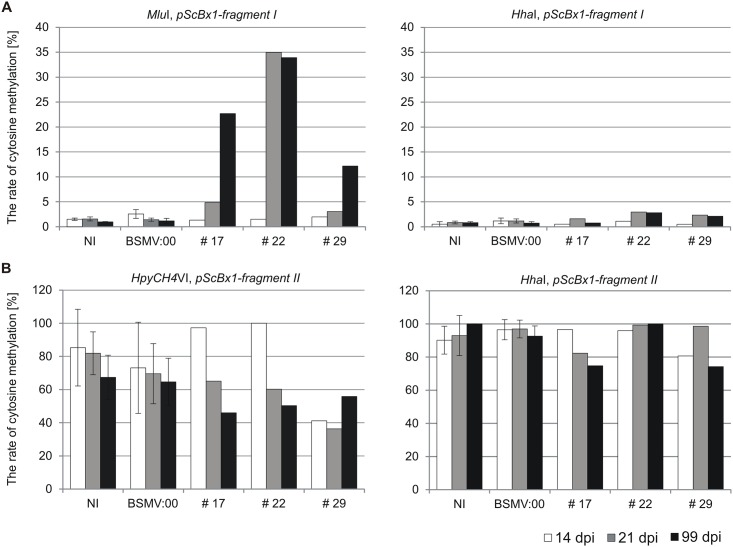
Rate of cytosine methylation in gDNA regions targeted with TGS vectors. Rate of cytosine methylation in sites recognized by *Mlu*I and *Hha*I in *fragment I* (**A**) and in sites recognized by *Hpy*CH4IV and *Hha*I in *fragment II* (**B**) of *ScBx1* promoter. NI—non-inoculated plants, BSMV:00 –plants inoculated with BSMV:α,β_(-)_,γ_(-)_ vector, #17, #22, #29 –plants inoculated with BSMV:α,β_(*pScBx1-fragment I*)_,γ_(*pScBx1-fragment II*)_ vector.

Genomic DNA corresponding to *fragment I* of the *ScBx1* promoter contains 3 sites recognized by MSRE: single *Mlu*I site and two *Hha*I. Genomic DNA corresponding to *fragment II* of the *ScBx1* promoter contains two *Hpy*CH4IV sites and a single *Hha*I site (Fig 5). The rate of cytosine methylation in all these sites has been analyzed. The rates of cytosine methylation in *fragment I Mlu*I site in non-inoculated plants were 1.5% (14 dpi), 1.6% (21 dpi) and 1.0% (99 dpi). The methylation rates in plants inoculated with the BSMV:00 vector were 2.5% (14 dpi), 1.4% (21 dpi) and 1.2% (99 dpi). The rates of methylation in the *Mlu*I site in plants inoculated with the experimental vector BSMV:*pScBx1* varied from 1.3% to 2.0% (14 dpi), from 4.8% to 35.0% (21 dpi) and from 2.0% to 12.2% (99 dpi) (Fig 7A, *Mlu*I, *pScBx1-fragment I*).

The rates of cytosine methylation in the *fragment I Hha*I site in non-inoculated plants were 0.5% (14 dpi), 0.9% (21 dpi) and 0.8% (99 dpi). The rates of methylation in plants inoculated with the BSMV:00 vector were 1.2% (14 dpi), 1.2% (21 dpi) and 0.7% (99 dpi). The rates of methylation in the *Hha*I site in plants inoculated with the BSMV:*fragment I* vector varied from 0.5% to 1.0% (14 dpi), from 1.6% to 3.0% (21 dpi) and from 0.8% to 2.8% (99 dpi) (Fig 7A, *Hha*I, *pScBx1-fragment I*).

The methylation rates in the *fragment II Hpy*CH4IV site in non-inoculated plants were 85.3% (14 dpi), 81.9% (21 dpi) and 67.3% (99 dpi). The methylation rates in BSMV:00 inoculated plants were 73% (14 dpi), 69.6% (21 dpi) and 64.6% (99 dpi). The methylation rates in the *Hpy*CH4IV site in plants inoculated with BSMV:*fragment II* vector (i.e. #17, #22, #29) varied from 41.2% to 100% (14 dpi), from 36.4% to 65.1% (21 dpi) and from 46% to 55.9% (99 dpi) (Fig 7B, *Hpy*CH4IV, *pScBx1-fragment II*).

The methylation rates in the *fragment II Hha*I site in non-inoculated plants were 90.2% (14 dpi), 93% (21 dpi) and 100% (99 dpi). The methylation rates in BSMV:00 inoculated plants were 96.5% (14 dpi), 96.9% (21 dpi) and 92.6% (99 dpi). The methylation rates of the *Hha*I site in plants inoculated with BSMV:*pScBx1-fragment II* vector (i.e. #17, #22, #29) ranged from 80.7% to 96.6% (14 dpi), from 82.4% to 99.3% (21 dpi) and from 74.2% to 100% (99 dpi) (Fig 7B, *Hha*I, *pScBx1-fragment II*).

### Elevated cytosine methylation in *ScBx1* promoter correlates with lower *ScBx1* transcript level and lower BX content

**Fig 8:**
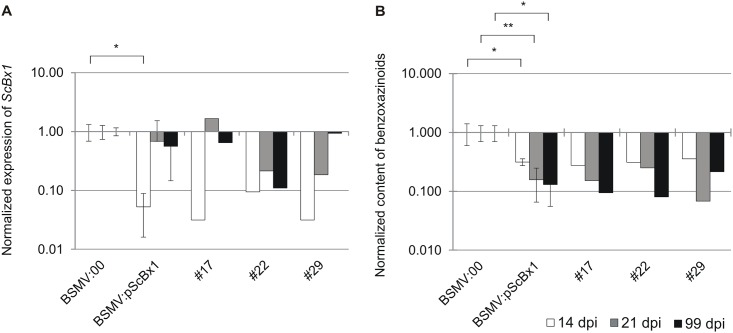
Normalized expression of *ScBx1* and normalized content of benzoxazinoids in TGS experiments. Normalized expression of *ScBx1* (**A**) and normalized content of benzoxazinoids (BXs) (**B**) in control plants (BSMV:00) compared to expression in experimental plants inoculated with BSMV:α,β_(*pScBx1-fragment I*)_,γ_(*pScBx1-fragment II*)_ (BSMV:*pScBx1*). Results are shown on a logarithmic scale. BSMV:00 –the mean value of *ScBx1* relative expression in plants inoculated with BSMV:α,β_(-)_,γ_(-)_ vector assumed as 1.00. BSMV:*pScBx1*—the mean value of *ScBx1* normalized expression in leaves inoculated with silencing vector. #17, #22, #29 –the *ScBx1* normalized expression in plants inoculated with silencing vector. Statistical significance (Student’s *t*-test): ** p < 0.005, * 0.005 < p < 0.05.

On average normalized expression of *ScBx1* in plants inoculated with the experimental vector (BSMV:*pScBx1*) compared to the control (BSMV:00) was 20 x lower at 14 dpi, 1.5 x lower at 21 dpi and 1.7 x lower at 99 dpi (Fig 8A). The strongest impact of inoculation with BSMV:*pScBx1* on relative expression of *ScBx1* was at 14 dpi (Fig 8A).

The normalized content of BXs in plants inoculated with the experimental vector (BSMV:*pScBx1*) compared to the control (BSMV:00) was 3.2 x lower at 14 dpi, 6.3 x lower at 21 dpi and 7.7 x lower at 99 dpi (Fig 8B). The normalized content of BXs in plants (#17, #22, #29) inoculated with BSMV:*pScBx1* compared to the control (BSMV:00) varied from 0.28 to 0.36 (14 dpi), 0.07 to 0.25 (21 dpi) and from 0.08 to 0.22 (99 dpi) (Fig 8B).

## Discussion

*Bx1*, the first gene of the BX biosynthesis pathway, encoding indole-3-glycerol phosphate lyase (IGL), was cloned for the first time in maize using the recessive *bx1* mutation [14]. This opened the possibility to identify *Bx1* orthologs in other cereal species: hexaploid wheat [20], wild barley [18], and diploid wheat species [19]. Putative *Bx* orthologs in rye were identified by La Hovary [35] and recently by Bakera *et al*. [23]. It is worthwhile to point that although the *benzoxazineless1* (*bx1*) mutation was originally used to identify and to clone *Bx1*, the gene itself and the encoded protein (BX1) are not the only ones responsible for synthesis of indole, which is the substrate for the BX biosynthesis pathway. Conversion of indole-3-glycerol phosphate to indole is catalyzed by enzymes encoded by at least three different genes: *Bx1*, *TSA* and *Igl*. All of the genes as well as the encoded proteins show a high level of sequence similarity, which is the result of the common evolution history [36, 38, 39]. The two *Bx1* and *Igl* genes encode enzymes that are active as monomers and can deliver free indole to the BX biosynthesis pathway [36]. The third one encodes the tryptophan synthase alpha subunit (TSA), which forms a complex with the tryptophan synthase beta subunit (TSB) and only in this configuration catalyzes indole synthesis [36, 37]. The IGL enzyme (encoded by the *Igl* gene) is active as a monomer and in this respect it can replace BX1 (encoded by the *Bx1* gene) in providing free indole for BX biosynthesis [18, 36]. The possible presence of two functionally redundant genes, which show a high level of nucleotide similarity, indicates that the final assignment of the putative *Bx1* gene should be the result of both *in silico* analysis and experimental verification.

A rye candidate of the *Bx1* ortholog has been isolated from the BAC library and assigned as the putative *ScBx1* gene based on its nucleotide sequence analysis [23]. Here the experimental characterization of the gene in rye is presented. The *ScBx1* transcript was detected only in young rye seedlings, indicating a strong, developmentally regulated expression pattern. The highest level, found in leaves 3 dai, was followed by a sharp decline; thus in leaves collected 21 dai the transcript was already undetectable. The expression pattern correlated with BX content. In non-induced seedlings the highest level of BXs matched the highest transcript level found 3 dai. In older leaves the content of benzoxazinoids declined, although it never reached the level of zero as it did for the *Bx1* transcript. In contrast to the undetectable transcript level, the content of BXs in rye leaves remained at low but still detectable levels throughout the whole analyzed period, i.e. until 99 dai. The seedling-specific expression of the analyzed gene corresponds well to expression patterns of *Bx1* orthologs found in rye [35], maize [12, 14], and wheat [22]. Based on these results we assume the presence of another gene encoding protein with IGL-enzyme activity, which acts redundantly with the *ScBx1*. We expect that the gene, which remains to be identified in rye, will be expressed at least in plants older than 21 dai.

Further results indicated strong activation of the putative *ScBx1* gene upon inoculation with the BSMV-based vector. The expression remained elevated during the whole analyzed period, i.e. 99 days after imbibition. The changes of *ScBx1* expression induced by the BSMV-based vector are similar to changes of *Bx1* orthologs caused by biotic stresses in other species: in maize upon herbivory infestation [4, 40, 41] or after treatment with stress-dependent plant elicitor peptide *ZmPep1* [42], after treatment with jasmonic acid (JA) or after pathogen and herbivory infection [43]. The experimentally determined expression pattern is in agreement with the presence of stress-specific motifs detected in the *ScBx1* promoter sequence [23]. The seedling-specific and stress-dependent expression of the gene correlates well with the content of BXs, as it has been confirmed by a high correlation coefficient between seedling-specific and BSMV-induced expression of *Bx1* with the accumulation of BXs.

Biological function of the putative *ScBx1* gene was analyzed using the virus-induced gene silencing (VIGS) strategy. The chosen method proved to be very effective in a number of plant species. The method is particularly useful in species where transformation-based tools are difficult to use or are not available (for a comprehensive review see [44]. Rye belongs to this group of recalcitrant plants. Although the species is a nonhost for *Barley stripe mosaic* v*irus* the presented results proved that the BSMV-based VIGS vector could be successfully used for gene silencing experiments. The inoculation rates were efficient and ranged from 4.7% to 12.5% as estimated from inoculations of 569 seedlings. Experimental inoculations were done with the *Bx1* silencing vectors containing fragments of the *PDS* marker gene and of the analyzed *Bx1* cloned alternatively to the β or γ subunit of the BSMV-based VIGS vector [30]. Both constructs resulted in similar inoculation efficiencies (10.4% and 9.3%) indicating that both β and γ subunits of the BSMV vector were equally suitable for the purpose. It is worth noting that the system of simultaneous posttranscriptional silencing of the two genes allowed for easy visual screening of photobleached plants, which presumably showed decreased expression of the analyzed gene. Only the plants showing successful inoculation with the BSMV-based vector were used for further molecular analysis. The experiments proved that *PDS* fragment conserved between barley and wheat was effective as a visual screening marker of VIGS in rye. The analysis of 9 plants confirmed that the *ScBx1* expression and the BX content were significantly lowered compared with control plants inoculated with the BSMV:*PDS* vector. Lowered BX content was highly correlated with decreased transcript level of the analyzed gene indicating strong functional association between *ScBx1* expression and BX biosynthesis. It is consistent with other examples of using VIGS as a reverse genetics tool. A similar approach of genes’ silencing was used to investigate regulation of morphine biosynthesis in opium poppy [33]. The results of BSMV-based silencing of *CYP96B22* encoding P450 monooxygenase was the basis to conclude about a role of the enzyme during basal resistance of barley against *Magnaporthe* [45]. Similar strategy was also applied to dissect contribution of glycerol-3-phosphate dehydrogenase and GLI1-encoded glycerol kinase in wheat systemic acquired resistance [46] and to establish function of zinc finger protein TaLSD1 in wheat resistance against stripe rust [47].

Opposite to the VIGS-based posttranscriptional silencing, there are relatively few reports on viral vector-induced transcriptional silencing (TGS) of endogenes in plants. It is partly because the endogenes were thought to be recalcitrant to the RNA-directed transcriptional silencing and most of the first attempts were focused on transgenes. Plant RNA viruses such as *Potato virus X* (*PVX*), *Tobacco rattle virus* (*TRV*) and *Cucumber mosaic virus* (*CMV*) vectors were used as tools to induce TGS of transgenes [48–50]. More recent studies, however, proved that endogenes can also be successfully targeted with virus-based vectors and consequently transcriptionally silenced. A high frequency of cytosine methylation at the targeted gene promoters was found in petunia and tomato plants inoculated with a *Cucumber mosaic virus* (*CMV*)-based vector [51]. Four *Arabidopsis* endogenes were silenced using a *Tobacco rattle virus RNA 2* (TRV2)-based vector [52]. Similar approach was used to confirm the role of cytosine methylation in promoter of *PcMYB10* in anthocyanin biosynthesis in pear fruits [53]. Novel insight on RNA-directed-DNA-methylation, the process which is the basis of virus-induced TGS is presented recently by Bond and Baulcombe (2015) [54].

The VIGS-BSMV vector reported here was constructed to accommodate the two different fragments derived from the *ScBx1* promoter region. Four sites recognized by three MSRE (*Mlu*I, *Hha*I and *Hpy*CH4IV) and located within the cloned regions served for analysis of cytosine methylation. Native cytosine methylation in the two target regions differed significantly. The methylation was low (0.5% to 1.6%) in both MSRE sites of the *pScBx1-fragment I* and it was much higher (67.3% to 100%) in *Hpy*CH4IV and *Hha*I sites of the *pScBx1-fragment II*. The level of native methylation was not affected in plants inoculated with the BSMV:00 vector. Methylation in plants inoculated with the experimental vector changed compared with the controls, although the degree was different depending on the analyzed region. Cytosine methylation in the *Mlu*I site of *pScBx1-fragment I* remained unchanged in plants 14 dpi and subsequently (21 and 99 dpi) significantly increased. In the *Hha*I site the methylation remained at a low level for the whole analyzed period. Cytosine methylation in the *Hpy*CH4IV site of the *pScBx1-fragment II* was high in the control plants and did not show any significant changes in experimental plants. Methylation of cytosine in the *Hha*I site was almost 100% in control and in experimental plants.

The significant increase of methylation in the *Mlu*I site of *pScBx1-fragment I* was the most probable cause of *ScBx1* silencing and consequently decreased levels of benzoxazinoids in rye leaves. The *Mlu*I site with induced cytosine hypermethylation is located in the 5′-UTR region of analyzed *ScBx1* 80 bp upstream of the ATG codon and the *pScBx1-fragment I* contains two TATA-box domains (Fig 5). According to [55] and [56] the close proximity (about 100 bp) of the induced methylation to transcription start site was crucial for RNA-directed DNA methylation and for transcriptional silencing. The native hypermethylation of the *pScBx1-fragment II* and no apparent effect on *Bx1* gene transcription in control plants agrees with the finding that methylation of more downstream regions of the promoter is unassociated with gene expression [55]. Similarly, the TGS approach to analyze function of the gene was used by Wang et al. (2013). The hypermethylation of the *PcMYB10* promoter found in green-skin pear was experimentally induced in corresponding hypomethylated DNA of red-skin pear. The result of *Agrobacterium tumefaciens* infiltration assay was methylation of the originally hypomethylated *PcMYB10* promoter transcriptional silencing of the *PcMYB10* gene and anthocyanin-free green-skin phenotype [53]. The results confirmed the role of *PcMYB10* promoter methylation in anthocyanin synthesis. It is worth to note that selected method allowed for a detection of methylation changes only within sites recognized by MSRE and the possible methylation in the neighboring regions could not be excluded. Bisulfite sequencing, the alternative to MSRE method, could not be applied because we expected that virus induced methylation pattern was not homogenous in all cells what might lead to ambiguous sequencing results.

The results presented here show that the analyzed *ScBx1* gene is functionally involved in benzoxazinoid biosynthesis in rye and its expression is developmentally regulated. Both traits, which also characterize *Bx1* orthologs in other monocots, confirm that the analyzed gene is a rye ortholog of the *Bx1* gene. Our results prove the applicability of the BSMV-based virus induced transcriptional and posttranscriptional silencing for functional analysis of rye genes. Moreover, based on the obtained results, we anticipate the presence of the *Igl* ortholog in rye, which contributes along with the *ScBx1* to BX biosynthesis in rye. We expect that the gene will be constitutively expressed at least in plants older than 21 dai. The gene remains to be identified.

## Materials and methods

### Plant material and growth conditions

Two rye (*Secale cereale* L.) hybrid cultivars, Stach F1 and Konto F1, were used. Kernels after 48 hours of imbibition in water-saturated filter paper were cultivated in pots (5 cm diameter) filled with Aura peat substrate (Lasland, Poland) mixed with sand in a 10:3 v/v ratio. The seedlings were cultivated in a growth chamber with a 16 h photoperiod, at 22°C in the day and 20°C at night. The relative humidity was in the range 60–80%, and the light intensity was 130–230 μM⋅s^-1^m^-2^. Hypocotyl and leaf samples were collected for transcript quantification and for hydroxamic acid analysis. The first leaves of 5-day old seedlings were used for inoculation with VIGS-BSMV vectors.

### Construction of VIGS-BSMV vectors

**Fig 9:**
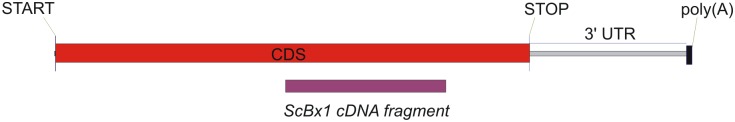
Schematic representation of cDNA (1290 bp) containing *ScBx1* gene (JQ716987.1). Indicated are START and STOP codon, CDS, 3′-UTR region and the fragment cloned to the VIGS-BSMV vector for posttranscriptional gene silencing (*ScBx1* cDNA fragment).

For VIGS studies the plasmids (pBSMV-T7-α, pBSMV-T7-γMCS and pBSMV-T7-γPDS275) carrying full-length cDNA clones of α and γ molecules of BSMV with the promoter of T7 RNA polymerase [57, 58] were kindly provided by Dr. Merete Albrechtsen (University of Aarhus, Frederiksberg, Denmark). The pT7-BSMV-β*Bam*HI plasmid carrying cDNA of the β molecule contained a unique *Bam*HI cloning site engineered between the open reading frame of βc (ORF βc) and poly(A) [30]. The plasmid pBSMV-T7-γMCS contained a multiple cloning site (MCS) with unique *Pac*I, *Xma*I, *Bam*HI, enabling cloning of DNA fragments upstream of the γb open reading frame. The plasmid pBSMV-T7-γ*PDS275* contained a 275-bp fragment of barley phytoene desaturase (*PDS*) encoding gene, accession number AY062039 [58]. The ‘empty’ BSMV:β*Bam*HI and BSMV:γMCS molecules were described as β_(-)_ and γ_(-)_ respectively. The β and γ molecules of pT7-BSMV-β*Bam*HI and pBSMV-T7-γMCS were modified by insertion of a cDNA fragment for posttranscriptional silencing and gDNA fragments for transcriptional silencing (S1 and S2 Tables, Figs 9 and 5). The fragments were amplified using primers (S1 Table) designed for cDNA (JQ716987) or gDNA (unpublished). The putative regulatory sequences of the *ScBx1* promoter were predicted using plant-dedicated SofBerry http://linux1.softberry.com/all.htm software. Based on the analysis the two fragments were selected. Both contained putative promoter specific motifs as well as the sites recognized by methylation-sensitive restriction enzymes (MSRE) needed for methylation analysis of the region. Amplicons after digestion with *Bam*HI and gel purification were ligated to the *Bam*HI site of the pBSMV-T7-β*Bam*HI and pBSMV-T7-γMCS plasmids. To confirm the presence of the insert the constructs were sequenced using primers designed for the insert-flanking region of pBSMV-T7 plasmids (S1 Table). Fragments of gDNA of the putative *ScBx1* promoter selected based on the presence of TATA-box motif and CG islands (Fig 5) (unpublished results) were used for construction of the BSMV:β and BSMV:γ subunits designed for transcriptional gene silencing. Genomic *fragment I* (324 bp), amplified with primers 2701_Fw and 2991_Re and genomic *fragment II* (313 bp) amplified with primers 1448_Fw and 1760_Re were cloned into BSMV:β*Bam*HI and BSMV:γMCS respectively (S2 Table, Fig 5).

### Nucleic acid isolation, reverse transcription and transcript quantification

Genomic DNA was isolated from young leaves according to the modified CTAB procedure [59]. The RNA was extracted from leaf samples using RNA 3-zone reagent (Novazym, Poland) according to the manufacturer’s instructions. RNA concentration and 260/A280 ratio were measured using a NanoDrop spectrophotometer (NanoDrop Technologies, USA). The 260/A280 ratio was always higher than 1.8 and agarose gel was used to further determine the quality of obtained RNA. Isolated total RNA was treated with 10 U of DNase (DNase I recombinant RNase-free, Roche, USA) and 10 U Protector RNase inhibitor [60] per 10 μg of RNA for 40 min at 37°C followed by DNase inactivation according to the manufacturer's protocol. Complete digestion was confirmed by PCR with primers specific for actin (AY145451) using 100 ng of DNase digested RNA as a template. No product on agarose gel was detected after 36 cycles of amplification. Four micrograms of RNA (non-degraded, DNase-treated with undetectable gDNA impurities) were used as a template for the reverse transcription reaction with oligo d(T) primers and the RevertAid First Strand cDNA Synthesis Kit (Thermo Scientific, USA). The obtained cDNA was diluted twofold and used directly as a template for quantitative PCR (qPCR). The standard qPCR reaction mix was composed of: 2 μL master mix 5x HOT FIREPol EvaGreen qPCR Mix Plus (Solis Biodyne), 0.2 μL primer F (10 μM), 0.2 μL primer R (10 μM), cDNA 1 μL and water to 10 μL. The reaction was performed in a Rotor-Gene 6000 model 5-plex thermocycler (Corbett). The efficiencies of amplification E for all primer pairs were in the range 0.9 to 1.00 and R^2^ 0.99875–1.00000. The specificity of amplification was verified by melting curve analysis. Template concentrations ranging from 10^2^ to 10^6^ copies of analyzed amplicon and 10^3^–10^7^ copies of the reference gene *HvAct* (AY145451.1) per reaction were used as the standards for qPCR. The resulting data were normalized to actin (AY145451.1) using the two standard curves method. Threshold line, Ct values, standard curves and relative quantifications were determined using the proprietary Rotor-Gene 6000 software v 1.7 supplied by the thermocycler manufacturer. The qPCR experiments were performed using cDNA from each analyzed plant with three technical repetitions each. The whole procedure of RNA isolation, reverse transcription, qPCR conditions and data analysis met the MIQE criteria outlined by Bustin *et al*. [61].

### In vitro transcription and plant inoculation with VIGS-BSMV-derived RNA

**Fig 10:**
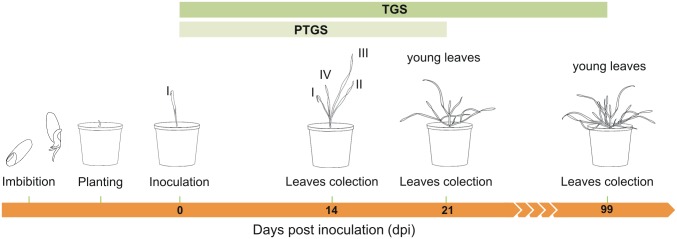
Timetable of VIGS experiment design. Inoculation of the first (I) leaf of 5 days old seedlings 0 dpi, collection of third (III) and fourth (IV) leaves with virus infection symptoms 14 dpi, collection of young leaves with virus infection symptoms 21 and 99 dpi.

The plasmids used as templates for *in vitro* transcription were isolated using the standard alkaline lysis method, linearized with *Mlu*I or *Spe*I (S2 Table) and purified according to the protocol by Kawalek *et al*. [30]. Resultant DNA, suspended in RNase free water, was used as template for *in vitro* transcription. Infectious RNA was synthesized using the *in vitro* transcription kit AmpliCap-Max T7 HighYield Message Maker Kit (CellScript, USA) according to the manufacturer’s protocol. A single *in vitro* reaction with 1 μg of plasmid-derived template yielded 30–40 μg of RNA. One μl of reaction mixture with approximately 2 μg of RNA of each subunit transcript (α, β and γ) was mixed with 18 μl of FES buffer (0.1 M glycine, 0.06 M K_2_HPO_4_, 1% Na_4_P_2_O_7_ (w/v), 1% Bentonite (w/v), 1% Celite (w/v)) [62]. The resulting 21 μl was rubbed into the leaf surface of 5-day-old rye seedlings dusted with silicon carbide (Silicon carbide 400 mesh, Sigma-Aldrich). The leaves of the control plants were powdered with silicon carbide and rubbed without or with FES buffer. Directly after the inoculation, the plants were transferred to a high humidity growth chamber (26°C, 100% relative humidity, 16 h photoperiod and 130–230 μM photons s^-1^m^-2^ light intensity). After 2 days, the plants were transferred to standard growth conditions. From several VIGS-BSMV inoculated plants only plants showing virus infection and/or *PDS* silencing (i.e. leaf photo bleaching) symptoms were selected for further studies. The leaves were collected 14 and 21 or 14, 21 and 99 days post inoculation (dpi) for further analysis of posttranscriptional or transcriptional silencing respectively (Fig 10). At least three plants for each vector / time point combination from two independent VIGS experiments were selected and analyzed. In order to show the silencing of the analyzed *Bx1* gene in the experimental plants the relative expression of the gene in plants inoculated with the BSMV vector (designated as BSMV:*PDS* or BSMV:00) was assumed as 1.00. This value was subsequently used as a basis to calculate the normalized expression of *ScBx1* in the experimental plants inoculated with the *ScBx1* silencing VIGS vectors. The outcome was presented on the logarithmic scale where values below the base line 1.00 indicated that the expression of *ScBx1* in experimental plants was lower than expression in plants inoculated with control BSMV:*PDS* or BSMV:00.

### Detection and quantification of cytosine methylation in the selected region of rye gDNA

Cytosine methylations in target regions of the genomic DNA *fragment I* and *fragment II* (Fig 5) was quantified using methylation sensitive restriction enzymes (MSRE) and the qPCR approach according to the procedure of *OneStep* qMethyl Kit (Zymo Research, USA). Briefly: 0.9 μg of gDNA isolated from control and experimental plants (i.e. inoculated with BSMV:α,β_(-)_,γ_(-)_ or BSMV:α,β_(*pScBx1-fragment I*)_,γ_(*pScBx1-fragment II*)_ respectively) were suspended in a restriction buffer. Each of them was split into a test and a reference sample and only the test sample was digested with MSRE. DNA from the test and reference samples served as qPCR templates for amplification with primers (2811_Fw/2994_Re and 1448_Fw/1760_Re, S1 Table) flanking the analyzed region. The difference between cycle threshold (Ct) values for test and reference DNA samples (i.e. ΔCt) was used to calculate the ratio of cytosine methylation according to the equation below [63]:

$$\text{Cytosine methylation ratio }\left(\%\right)\;=\;100\text{ x }2^{-\Delta \text{Ct}}$$

### Sample preparation and analysis of selected benzoxazinoids

100 mg of lyophilized leaf samples were mixed with diatomaceous earth (ASE Prep DE, Dionex, Sunnyvale, CA) and extracted with 70% methanol in stainless steel extraction cells of an accelerated solvent extraction system (ASE 200, Dionex, Sunnyvale, CA). Extractions were carried out at 10 MPa operating pressure and 40°C. After evaporation to dryness under reduced pressure, extracts were reconstituted in 1 ml of methanol containing 0.1% (v/v) acetic acid and stored at -20°C. Before the analyses, extracts were centrifuged for 20 min at 23 000 x g at 4°C. Quantitative analyses were performed on a Waters Acquity UPLC system (Waters, Milford, MA) equipped with a triple quadrupole mass spectrometer (Waters TQD). BXs and their metabolites were separated on a Waters BEH C18 column (50 x 2.1 mm, 1.7 μm) with a linear, 7 min long gradient from 3 to 10% of acetonitrile containing 0.1% (v/v) formic acid (solvent B) in 0.1% formic acid (solvent A). All separations were carried out at 50°C at the flow rate of 700 μl∙min^-1^. After each gradient elution, the column was washed with 90% solvent B for 1 minute and then re-equilibrated with 3% solvent B in solvent A for 3 minutes prior to the next injection. Injections were done in the “partial loop needle overfill” mode of a Waters Acquity autosampler. 2.5 μl was injected from each sample and the analysis was repeated twice. The column’s effluent was introduced into the ion source of the mass spectrometer, which operated in the negative ion mode with the following parameters: capillary voltage -2.8 kV, extractor 3 V, RF lens 100 mV, source temperature 130°C, desolvation temperature 400°C, desolvation gas flow 1000 l∙h^-1^, cone gas flow 100 l∙h^-1^. The collision cell entrance and exit were set to -2 and 0.5, respectively. Parameters of quadrupoles 1 and 3 were set to achieve unit-mass resolution. Cone voltage and collision energy were optimized for each compound to attain a maximal response (Table 3).

**Table 3:** Parameters of mass spectrometer analysis.

Compound		RT [min]	*m/z* of parent [M-H]^-^ ion	*m/z* of fragment ion	Collision Energy [eV]	Cone voltage [V]
HBOA	2-hydroxy-4H-1,4-benzoxazin-3-one	2,10	164	108	15	30
DIBOA	2,4-dihydroxy-1,4-benzoxazin-3-one	2,30	180	134	6	20
DIBOA-Glc	4-hydroxy-2-[(3R,4S,5S,6R)-3,4,5-trihydroxy-6-(hydroxymethyl)oxan-2-yl]oxy-1,4-benzoxazin-3-one	2,80	342	162	15	25
DIMBOA	2,4-dihydroxy-7-methoxy-1,4-benzoxazin-3-one	3,70	210	149	6	15
DIMBOA-Glc	4-hydroxy-7-methoxy-2-[(3R,4S,5S,6R)-3,4,5-trihydroxy-6-(hydroxymethyl)oxan-2-yl]oxy-1,4-benzoxazin-3-one	4,60	372	149	15	35
MBOA	6-methoxy-3H-1,3-benzoxazol-2-one	5,20	164	149	15	20

The quantitation method was calibrated from the standard solutions of DIBOA, DIMBOA, HBOA, DIMBOA-Glc and MBOA. DIMBOA-Glc was used as a reference standard for DIBOA-Glc quantitation. Calibration was performed between 2.5 and 30 ng∙μl^-1^, and it was found to be linear only within this range. Samples with concentrations of analytes higher than 30 ng∙μl^-1^ were appropriately diluted (typically between 2 and 5 times) using 0.1% formic acid and re-analyzed. Waters MassLynx 4.1 SCN 849 software was used for data acquisition and processing.

### Statistical analysis

The statistical significance of the change (Student’s *t*-test) was calculated as * p < 0.05 and ** p < 0.005. Pearson’s coefficient was used to determine whether there was a relationship in the expression of *ScBx1* and benzoxazinoid content. The statistical analyses were performed using the software package STATISTICA (Data Analysis Software System, StatSoft Inc.). All quantitative results of cytosine methylation, transcripts and BXs levels represented medium value from three technical repetitions of single individual plant inoculated with BSMV-based vector.

## Supporting information

S1 Table List of primers and reaction conditions. Shading indicates *Bam*HI recognition site used for amplicon cloning.
(DOCX)

S2 Table List of the pBSMV-T7 plasmids. (DOCX)

S3 Table Amount of selected benzoxazinoids in rye hypocotyls 1, 2 and 3 days after imbibition and leaves 4, 7, 14 and 99 dai. The results represent mean values and standard deviations quantified in three independent biological replicates.
(DOCX)

S1 Fig Correlation analysis between relative expression of *ScBx1* and content of benzoxazinoids in rye hypocotyls/leaves collected 1, 2, 3, 4, 7, 14, 21 and 99 days after imbibition. The correlation coefficient value is 0.83 for p < 0.05 and N = 24.
(TIF)

S2 Fig Correlation analysis between relative expression of *ScBx1* and the content of benzoxazinoids in rye leaves collected 14 days post inoculation with BSMV-VIGS vector. Correlation coefficient = 0.85, p = 0.008.
(TIF)

S3 Fig Correlation analysis between relative expression of *ScBx1* and the content of benzoxazinoids in rye leaves collected 21 days post inoculation with BSMV-VIGS vector. Correlation coefficient = 0.57, p = 0.13.
(TIF)
