# Supplementary material for: Identification and VIGS-based characterization of Bx1 ortholog in rye (Secale cereale L.)
Source: PLoS One. 2017 Feb 24;12(2):e0171506. doi: 10.1371/journal.pone.0171506 (PMC5325281; doi:10.1371/journal.pone.0171506)
Supplement: S1 Table — Shading indicates BamHI recognition site used for amplicon cloning. (DOCX) [file pone.0171506.s001.docx]

S1 Table. List of primers and reaction conditions. Shading indicates *Bam*HI recognition site used for amplicon cloning.

| **Amplified template** | **Primer symbol** | **Sequence 5’ – 3’** | **Amplicon length [bp]** | **Conditions of qPCR** |
| --- | --- | --- | --- | --- |
| *ScBx1* | 467_Fw | ATGGATCCTCTTCTCGTACTACAGGCCCATC | 324 bp | 60°C |
|  | 791_Re | ATGGATCCAACAGCAACAGGTTTGTCAG |  |  |
| *pScBx1-fragment II* | 1448_Fw | ATGGATCCTTGAATAGCGCCATCATCACA | 313 bp | 63°C |
|  | 1760_Re | ATGGATCCCGCCTATATACTTACGTCATCCTCA |  |  |
| *pScBx1-fragment I* | 2701_Fw | ATGGATCCAATCTTTGGATCGGTCCACC | 297 bp | 63°C |
|  | 2991_Re | ATGGATCCACGATACATGAGTTGCGCGT |  |  |
| *ScBx1* | 2811_Fw^1^ | TGCTGGTGTTTGGTTGTATGC | 183 bp | 63°C |
|  | 2994_Re^1^ | ATACATGAGTTGCGCGTCTG |  |  |
| BSMV:β | Bins_Fw^2^ | ATGTGGGGAGGTTTAGTCAG | sequencing primers | insert cloned to the β subunit |
|  | Bins_Re^2^ | GAAGAAGATGCAGGAGCTGAA |  |  |
| BSMV:γ | Gins_Fw^2^ | GAAGAAGATGCAGGAGCTGAA | sequencing primers | insert cloned to the γ subunit |
|  | Bins_Re^2^ | CACTCCCATCATATGGTTGAT |  |  |
| *ScBx1* | q802_Fw^3^ | TCAAAACCTGAACACGTGAAGC | 124 bp | 60°C |
|  | q926_Re^3^ | GCCTCTAGCCTTTTCAATCCTTC |  |  |
| *HvAct* | qAct_Fw^3^ | AGCAACTGGGATGACATGGA | 172 bp | 60/62°C |
|  | qAct_Re^3^ | CGTACATGGCAGGAACATTG |  |  |

^1^ – primers used for methylation analysis of *ScBx1* promoter region

^2^ – primers used for insert sequencing

^3^ – primers used for qPCR transcript quantification
