# Supplementary material for: Identification and VIGS-based characterization of Bx1 ortholog in rye (Secale cereale L.)
Source: PLoS One. 2017 Feb 24;12(2):e0171506. doi: 10.1371/journal.pone.0171506 (PMC5325281; doi:10.1371/journal.pone.0171506)
Supplement: S2 Table — (DOCX) [file pone.0171506.s002.docx]

**S2 Table.** List of the pBSMV-T7 plasmids.

| **Plasmid** | **Restriction enzyme used for plasmid linearization** | **Symbol of BSMV-RNA obtained from pBSMV-T7 plasmid as the result of *in vitro* transcription** | **Remarks** |
| --- | --- | --- | --- |
| pBSMV-T7 | *Mlu*I | α, | α subunit, unmodified |
| pT7-BSMV-β*Bam*HI | *Spe*I | β_(-)_ | β subunit without insert, ‘empty’ |
| pBSMV-T7-*PDS*275 | *Spe*I | β_(_*_PDS_*_)_ | β subunit with fragment of *HvPDS* cDNA |
| pT7-BSMV-β*ScBx1* | *Spe*I | β_(_*_ScBx1_*_)_ | β subunit with fragment of *ScBx1* cDNA |
| pT7-BSMV-β *pScBx1-fragment I* | *Spe*I | β_(_*_pScBx1-fragment I_*_)_ | β subunit with gDNA fragment I derived from *ScBx1* promoter |
| pBSMV-T7-γMCS | *Mlu*I | γ_(-)_ | γ subunit without insert, ‘empty’ |
| pBSMV-T7-PDS275 | *Mlu*I | γ_(_*_PDS_*_)_ | γ subunit with fragment of *HvPDS* cDNA |
| pT7-BSMV- γ*ScBx1* | *Mlu*I | γ_(_*_ScBx1_*_)_ | γ subunit with fragment of *ScBx1* cDNA |
| pT7-BSMV- γ *pScBx1-fragment II* | *Mlu*I | γ_(_*_pScBx1-fragment II_*_)_ | γ subunit with gDNA fragment II from *ScBx1* promoter |
