# Supplementary material for: Identification and VIGS-based characterization of Bx1 ortholog in rye (Secale cereale L.)
Source: PLoS One. 2017 Feb 24;12(2):e0171506. doi: 10.1371/journal.pone.0171506 (PMC5325281; doi:10.1371/journal.pone.0171506)
Supplement: S3 Table — The results represent mean values and standard deviations quantified in three independent biological replicates. (DOCX) [file pone.0171506.s003.docx]

**S3 Table.** Amount of selected benzoxazinoids in rye hypocotyls 1, 2 and 3 days after imbibition and leaves 4, 7, 14 and 99 dai. The results represent mean values and standard deviations quantified in three independent biological replicates.

| **Days after imbibition** | **HBOA*** | **DIBOA*** | **DIBOA-Glc*** | **DIMBOA*** | **DIMBOA-Glc*** | **MBOA*** |
| --- | --- | --- | --- | --- | --- | --- |
| 1 | 70.3 / ±33.1 | 1082.5 / ±348.8 | 1279.8 / ±505.7 | 0.0 | 15.7 / ±8.1 | 0.0 |
| 2 | 252.1 / ±5.7 | 6402.0 / ±1743.5 | 119.6 / ±64.1 | 0.0 | 0.0 | 0.0 |
| 3 | 393.2 / ±26.3 | 9210.2 / ±1859.0 | 47.1 / ±12.1 | 0.0 | 0.0 | 40.6 / ±4.3 |
| 4 | 340.1 / ±26.4 | 6451.7 / ±1151.4 | 29.7 / ±5.8 | 0.0 | 0.0 | 43.9 / ±19.9 |
| 7 | 253.2 / ±3.2 | 3487.6 / ±498.7 | 18.4 / ±2.3 | 0.0 | 0.0 | 41.2 / ±11.1 |
| 14 | 124.1 / ±62.6 | 1152.5 / ±246.7 | 5.8 / ±0.6 | 3.9 / ±0.7 | 0.0 | 16.6 / ±8.9 |
| 21 | 95.4 / ±40.5 | 1011.0 / ±238.1 | 5.5 / ±0.5 | 4.1 / ±1.1 | 0.0 | 12.2 / ±9.1 |
| 99 | 24.9 / ±14.4 | 376.8 / ±227.2 | 4.1 / ±2.5 | 3.8 / ±2.9 | 0.0 | 3.3 / ±2.1 |

* - µg∙g^-1^ dry weight / SD values
